# The Influence of Liver Transplant on Serum Cholinesterase Levels: A Case Report

**DOI:** 10.7759/cureus.33761

**Published:** 2023-01-14

**Authors:** George Stancu, Elena Laura Iliescu

**Affiliations:** 1 Gastroenterology and Hepatology, Valahia Medical Center, Ploiesti, ROU; 2 Department of Internal Medicine, Fundeni Clinical Institute, Bucharest, ROU

**Keywords:** liver function, liver failure, serum cholinesterase, liver transplant recipients, alcohol related cirrhosis

## Abstract

Cholinesterase is a serum enzyme synthesized mainly by the hepatocyte. Serum cholinesterase levels tend to decrease in time in patients with chronic liver failure and can indicate the intensity of liver failure. The lower the value of serum cholinesterase, the higher the possibility of liver failure. A reduction in liver function induced a drop in the level of serum cholinesterase. We present a patient with end-stage alcoholic cirrhosis and severe liver failure that received a liver transplant from a deceased donor. We compared blood tests and serum cholinesterase before and after the liver transplant. The hypothesis is that the value of serum cholinesterase increases after a liver transplant, and we noticed a significant increase in cholinesterase levels after the transplant. Serum cholinesterase activity increases after a liver transplant and it indicates that the liver function reserve will reach a higher level after the transplant, according to the new liver function reserve.

## Introduction

Serum cholinesterase is an affordable, easy-to-obtain blood test that can estimate the liver reserve [1]. It is not affected by albumin and plasma treatments or by warfarin treatments that can influence Child-Pugh and Model for End-Stage Liver Disease (MELD) scores [2,3].

A liver transplant is the last treatment option for end-stage liver disease patients. In the case of a liver transplant, an increase in the serum cholinesterase activity will confirm the clinical value of this test.

We followed one patient admitted to the internal medicine ward of the Fundeni Clinical Institute. The patient received a deceased donor liver transplant for end-stage alcoholic cirrhosis. We obtained informed consent and performed clinical examinations, abdominal ultrasound, blood tests, and serum cholinesterase dosing at the Fundeni Clinical Institute. The liver transplant was also performed at our institution. We obtained blood tests and serum cholinesterase levels before and after the liver transplant. After the procedure, we followed the patient for five years and repeated the blood tests, and examined the cholinesterase levels.

## Case presentation

We selected a male patient with end-stage alcoholic liver cirrhosis that received a whole liver transplant. The patient was 60 years old at the moment of the transplant. The clinical examination identified jaundice, a cirrhotic liver and enlarged spleen, and grade 3 hepatic encephalopathy. The blood test showed mild anaemia and thrombocytopenia, prolonged coagulation tests, low albumin, mildly elevated transaminases, and liver function tests (Child-Pugh and MELD-Na score) with scores compatible with severe liver failure (Child C). All the parameters indicated end-stage liver disease and he urgently needed a liver transplant. The value of serum cholinesterase before the transplant was in accordance with the usual scores with a very low value of 2700 U/L (Table 1).

**Table 1:** Patient characteristics BCHE, cholinesterase; Leu, white blood cells; Plt, platelets; AP, prothrombin activity; INR, International, Normalised Ratio; ALAT, alanil amino transferase; ASAT, aspartat amoni transferase; GGT, gamma glutamyl transferase; Na, natrium;

Parameter	Before liver transplant	After liver transplant
Age (years)	60,00	65,00
Cholinesterase BCHE (U/L)	2700,00	7634,00
Hb (g/dL)	10,10	14,50
Leu (x10^3/µL)	4,60	4,58
Plt (x10^3/µL)	51,00	90,00
AP (%)	36,00	69,00
INR	2,14	1,28
Fibrinogen (mg/dL)	194,00	351,00
Albumin (g/L)	2,40	4,49
ALAT (U/L)	63,00	47,00
ASAT (U/L)	105,00	40,00
Total Bilirubin (mg/dL)	10,10	0,90
Direct bilirubin (mg/dL)	9,30	0,30
GGT (U/L)	36,00	7,00
FA (U/L)	141,00	52,90
Creatinine (mg/dL)	1,27	1,25
Na serum (mmol/L)	133,00	145,91
Encefalopaty grade	3,00	0,00
Ascites (ultrasound)	absent	absent
Child-Pugh score	12 pts (Child C)	5 pts (Child A)
Meld-Na Score	28 pts	11 pts

The liver transplant was performed and the patient survived. We monitored the patient and after five years we evaluated him again using the same procedures and blood tests (Table 1).

## Discussion

In patients with end-stage liver disease, the only option for treatment is a liver transplant. The procedure can prolong life expectancy. After the transplant, the new liver starts to synthesise proteins at an increased level. The serum proteins increase and the Child-Pugh score and MELD-Na score are influenced by the new values. The liver transplant of our patient had a positive effect on the liver function scores and the serum cholinesterase level. The Child-Pugh score improved from 12 points (Child C class) to 5 points (Child A class) and the MELD-Na score dropped from 28 points to 11 points. These scores showed that the liver function normalized (Table 1). 

The serum cholinesterase also increased from 2700 U/L to 7634 U/L. The value of cholinesterase after the transplant was in the normal range. This evolution of scores was also consistent with our hypothesis that a liver transplant increases the value of serum cholinesterase. We also noticed improvements in serum albumin levels, INR, prothrombin activity, haemoglobin, fibrinogen, transaminase, total bilirubin, and serum sodium levels. The normalisation of the liver function reserve determined the variations of all these parameters. 

This increase in the serum cholinesterase level after the liver transplant confirms that cholinesterase is an important tool in evaluating the liver function reserve and it can easily replace Child-Pugh and MELD-NA scores because it has a very low cost, and is available in all emergency departments. It also can be used in cirrhotic patients treated with frozen plasma, albumin, or warfarin, situations that make the Child-Pugh and MELD-NA scores unusable.

## Conclusions

This study demonstrates that the value of serum cholinesterase increases after a liver transplant. The improvements observed in the Child-Pugh Score and MELD-Na score confirm that the liver reserve increases after the liver transplant. The new liver is responsible for the increase in the liver function reserve. Serum cholinesterase can be a useful tool in evaluating the liver functional reserve before and after a liver transplant.
